# Cerebellar presence of immune cells in patients with neuro-coeliac disease

**DOI:** 10.1186/s40478-023-01538-5

**Published:** 2023-03-25

**Authors:** Maxine D. Rouvroye, Hetty J. Bontkes, John G. J. M. Bol, Birgit Lissenberg-Witte, Valerie Byrnes, Fadel Bennani, Ekaterina S. Jordanova, Micha M. M. Wilhelmus, Chris J. Mulder, Paul van der Valk, Annemieke J. M. Rozemuller, Gerd Bouma, Anne-Marie Van Dam

**Affiliations:** 1 Department of Gastroenterology and Hepatology, AGEM Research Institute, Amsterdam UMC, Vrije Universiteit Amsterdam, Amsterdam, The Netherlands; 2 Department of Anatomy and Neurosciences, Amsterdam Neuroscience, Amsterdam UMC, Vrije Universiteit Amsterdam, De Boelelaan 1108, 1081 HZ Amsterdam, The Netherlands; 3 Medical Immunology Laboratory, Department of Clinical Chemistry, Amsterdam Infection and Immunity Institute, Amsterdam UMC, Vrije Universiteit Amsterdam, Amsterdam, The Netherlands; 4 Department of Epidemiology and Data Science, Amsterdam UMC, Vrije Universiteit Amsterdam, Amsterdam, The Netherlands; 5 Department of Gastroenterology and Hepatology, Galway University Hospitals, Galway, Ireland; 6 Department of Pathology, Mayo University Hospital, National University of Ireland Galway Affiliated Hospital, Galway, Ireland; 7 Department of Gynecology and Obstetrics, Center for Gynecologic Oncology Amsterdam, Amsterdam UMC, Vrije Universiteit, Amsterdam, The Netherlands; 8 Department of Pathology, Amsterdam UMC, Vrije Universiteit Amsterdam, Amsterdam, The Netherlands; 9 Present Address: Department of Gastroenterology and Hepatology, Spaarne Gasthuis, Boerhavelaan 22, 2035 RC Haarlem, The Netherlands

**Keywords:** Gluten, Coeliac disease, Neurological disorders, Ataxia, Encephalopathy, Lymphocyte, Purkinje cell, Microglia, Immunohistochemistry, Cerebellum

## Abstract

Although various neurodegenerative disorders have been associated with coeliac disease (CD), the underlying neuropathological link between these brain and gut diseases remains unclear. We postulated that the neuronal damage sporadically observed in CD patients is immune-mediated. Our aim was to determine if the loss of neurons, especially Purkinje cells, coincides with microglia activation and T- and B-cell infiltration in the cerebellum of patients with CD and a concomitant idiopathic neurological disease affecting the cerebellum (NeuroCD). Post-mortem cerebellar tissue was collected of validated NeuroCD cases. Gender- and age-matched genetic spinocerebellar ataxia (SCA) controls and non-neurological controls (NNC) were selected based on clinical reports and pathological findings. Cerebellar tissue of seventeen patients was included (6 NeuroCD, 5 SCA, 6 NNC). In SCA cases we found that the Purkinje cell layer was 58.6% reduced in comparison with NNC. In NeuroCD cases this reduction was even more prominent with a median reduction of 81.3% compared to NNC. Marked increased numbers of both CD3+ and CD8+ cells were observed in the NeuroCD but not in SCA patients. This coincided with significantly more microglial reactivity in NeuroCD patients. These findings demonstrate that the massive loss of Purkinje cells in the cerebellum of neuro CD patients is accompanied by local innate and T-cell mediated immune responses.

**Supplementary Information:**

The online version contains supplementary material available at 10.1186/s40478-023-01538-5.

## Introduction

Coeliac disease (CD) is a systemic disorder caused by an immune-mediated response to dietary gluten in genetically susceptible individuals, hallmarked by an enteropathy [1]. This enteropathy is characterised by villous atrophy, crypt hyperplasia and an increased population of intraepithelial lymphocytes (IELs) in the duodenal mucosa [1]. These IELs play a central role in the induction of apoptosis of epithelial cells by means of Granzyme B and perforin secretion [2]. Strict adherence to a gluten-free diet can reverse the intestinal damage. In incidental cases enteropathy and malabsorption persist or recur despite strict dietary adherence [3]. In some of these patients, referred to as refractory coeliac disease type 2 (RCD2), clonal expansion of a population of lineage negative cells with an unusual or ‘aberrant’ phenotype is found. Although extremely rare, RCD2 has a severe disease course and may transform into an enteropathy associated T-cell lymphoma [4–6].

A small number of biopsy confirmed CD patients has been reported in the literature who developed otherwise idiopathic neurological manifestations in the absence of nutrient deficiencies [7]. The most common neurological symptoms in people with CD or gluten sensitivity are ataxia and neuropathy while several other neurological manifestations have been reported in the context of CD. We refer here to the general term NeuroCD for those patients with biopsy confirmed CD. Such neurological symptoms have also been described in patients that do not meet the diagnostic criteria for CD, but do exhibit manifestations of serological immune reactivity to gluten related peptides, referred to as gluten ataxia [8–11]. A prospective assessment of 1500 ataxia patients resulted in a diagnosis of gluten ataxia in 20% of cases. The diagnosis of gluten ataxia was based on the presence of serologically low-titre gluten-related antibodies (e.g. anti-gliadin antibodies (AGA), regardless of anti-Transglutaminase 2 antibodies or duodenal histology) [10]. An improvement in ataxia scores and subjective clinical improvement was demonstrated after initiation of a gluten-free diet [12], suggesting a link between dietary gluten intake and the development of such neurological manifestations.

The underlying pathophysiological mechanism for gluten related neurological manifestations is unclear, although an immune-mediated mechanism is suspected [13]. Thus far, only a few case-reports have been published on post-mortem observations in NeuroCD patients demonstrating a loss of cerebellar Purkinje cells and signs of inflammation, e.g. infiltrating leukocytes [14–16]. In the present study, we aimed to elaborate on these observations and further define the presence and localization of immune cells in the cerebellum. Hereto, we established a detailed, semi-quantitative immunohistochemical analysis of the cerebellum of a cohort of NeuroCD cases that we compared to gender- and age-matched patients with a genetic cause of ataxia (SCA) and non-neurological control subjects (NNC).

## Methods

### Human post-mortem material

Post-mortem tissue of patients with an established diagnosis of CD and concomitant idiopathic (cerebellar) neurodegeneration, defined as NeuroCD, was collected from the Netherlands Brain Bank (NBB) Amsterdam, The Netherlands (www.brainbank.nl) (case NeuroCD6), the VU university medical centre biobank (case NeuroCD1), the University Medical Centre Utrecht biobank (cases NeuroCD2 to 4) and the University College Hospital Galway (case NeuroCD5). Brain material of the NeuroCD cases used have not been studied before or elsewhere. Tissue of 6 gender- and age-matched control subjects without neurological or psychiatric disease (cases NNC1 to NNC6) and of 5 cases with a genetic form of SCA presenting loss of Purkinje cells (cases SCA1 to SCA5), was obtained from NBB. In compliance with all local ethical and legal guidelines, informed consent for brain autopsy and the use of brain tissue and clinical information for scientific research was given by either the donor or the next of kin. Clinical information of patients from which brain material was used in this study, is provided in Table 1.

**Table 1:** Patient characteristics

Subject	Gender	Age at diagnosis of CD	CD	Age at analysis neurological complaints	Age of death	Neurological disease	Cause of death
*NeuroCD*							
1	m	49	RCD2 + EATL	64	64	Encephalitis	Encephalitis
2	f	58	RCD2	58	58	Encephalopathy + epilepsy	renal failure + sepsis
3	f		CD	69	71	Cerebellar syndrome + myoclonus	Severe neurodegeneration
4	m		CD	75	77	Cerebellar syndrome + myoclonus	Cachexia, Pneumonia
5	f	52	RCD2	52	74	Cerebellar syndrome	Central line infection
6	f	47	CD	51	64	progressive ataxia + myoclonus	respiratory insufficiency
*SCA*							
1	m	n/a	–	31	82	Spinocerebellar ataxia	Respiratory insufficiency
2	f	n/a	–	74	82	Spinocerebellar ataxia	cachexia, dehydration
3	f	n/a	–	70	86	Spinocerebellar ataxia	cachexia, aspiration pneumonia
4	f	n/a	–	65	71	Spinocerebellar ataxia	Cachexia and dehydration
5	m	n/a	–	42	63	Spinocerebellar ataxia	Euthanasia
*NNC*							
1	m	n/a	–	n/a	65		Cardiac arrest
2	f	n/a	etc	n/a	66		Terminal heart failure
3	m	n/a		n/a	77		Perforation of the bladder (malignancy)
4	f	n/a		n/a	61		Euthanasia (ovary cancer)
5	m	n/a		n/a	55		Euthanasia (oesophageal cancer)
6	f	n/a		n/a	72		Euthanasia (ovary cancer)

In this table the patient characteristics per patient per group are displayed with gender, age at time of diagnosis of coeliac disease, age at time of analysis of neurological symptoms and age of death. Their neurological syndrome and probable cause of death. Please see the Additional file 1 for additional clinical and pathological information

*NeuroCD* Neuro-coeliac disease, *SCA* Genetic spinocerebellar ataxia, *NNC* Non-neurological controls, *m* Male, *f* Female. *CD* Coeliac disease, *RCD2* Refractory coeliac disease type 2, *EATL* Enteropathy associated T-cell lymphoma, *n/a* Not applicable

### Immunohistochemistry

After autopsy, dissected cerebellar tissue was fixed in 4% formalin and subsequently embedded in paraffin. From the obtained paraffin blocks, 7 μm sections were cut on a microtome and mounted on positively charged glass slides (Permafrost) and incubated on a heated plate for 1 h at 43 °C. Afterwards, slides were dried overnight in an incubator at 37 °C before being stored at room temperature (RT). Upon use for immunohistochemistry, tissue sections were heated to 58 °C for 30 min. Subsequently, sections were deparaffinized in xylene (100%) and graded ethanol series (100, 96, 80 and 70%) to demi-water. CD3, CD8, CD20, Granzyme B and Iba-1 antibody immunohistochemical stainings were performed at the department of Pathology (Amsterdam UMC) using the Ventana Immunostainer (Ventana Medical System, Arizona, USA). All stainings were performed according to the manufacturer’s instructions, summarised in Tables 2, 3, and counterstained with hematoxylin. The Glial Fibrillary Acidic Protein (GFAP), Calbindin and TMEM119 immunohistochemical antibody stainings were performed manually at the department of Anatomy and Neurosciences (Amsterdam UMC). For the latter stainings, antigen retrieval was performed by heating the sections in a 10 mM Tris-EDTA (pH 9.0) solution that was pre-heated in a microwave before placing it in a steam cooker for 30 min and left to cool down to RT. Sections were then washed with Tris-buffered saline (TBS) (pH 7.6) and endogenous peroxidase was blocked with 1% H_2_O_2_ in TBS for 30 min. After a short wash in TBS, the were incubated in TBS containing 0.5% Triton (TBS-T) and 5% non-fat dry milk (Campina, Netherlands; block buffer) for another 30 min to reduce non-specific antibody binding. Subsequently, sections were incubated overnight with Calbindin-, GFAP- or TMEM119 antibody in the same block buffer (see Tables 2, 3 for details). The next day, the sections were thoroughly washed in TBS and incubated for 2 h at RT in block buffer containing corresponding biotinylated IgG’s according to Tables 2, 3. Afterwards, the sections were washed in TBS and incubated for 1 h with horseradish peroxidase labeled avidin–biotin complex (ABC complex, 1:400, Vector Labs) in TBS-T at RT. Finally, after washes in TBS and Tris-HCl, immunoreactivity was visualized by adding 3,3-diaminobenzidine (DAB, Sigma, St. Louis, USA). All sections were counterstained with haematoxylin. Sections were dehydrated through a graded series of alcohol, cleared in xylene and mounted with Entellan mounting medium (Merck Millipore, Darmstadt, Germany).

**Table 2:** Antibodies used for manually performed stainings

Antigen	Antigen Retrival	Species	Dilution	Manufacturer	Article number	Secondary antibody	Manufacturer	Article number	Dilution
GFAP	Tris/EDTA Ph9.0	Rabbit polyclonal	1:4000	DAKO	Z0334	GAR-biot	Jackson	111–065-144	1:400
Calbindin	Tris/EDTA Ph9.0	mouse monoclonal	1:400	Swant	300	GAM-biot	Jackson	115–065-146	1:400
TMEM119	Tris/EDTA Ph9.0	Rabbit polyclonal	1:500	Atlas	HPA051870	GAR-biot	Jackson	115–065-146	1:400
CD3*	Tris/EDTA Ph9.0	Rabbit polyclonal	1:50	DAKO	A0452	Alexa 546, Isotype GAR	Life Technologies, Thermo Fisher scientific	A11010	1:200
CD8*	Tris/EDTA Ph9.0	Mouse monoclonal	1:75	Novocastra, Leica Biosystems	ncl-l-cd8-4b11	Alexa 647, Isotype GAM IgG2b	Life Technologies, Thermo Fisher scientific	A21242	1:200
Granzyme B*	Tris/EDTA Ph9.0	Mouse IgG2a	1:100	Invitrogen, Thermo Fisher scientific	MA1-35,461	A488 Isotype GAM IgG2a	Life Technologies, Thermo Fisher scientific	A21131	1:200

**Table 3:** Antibodies used for Ventana performed stainings

Antigen	Manufacturer	Article number	Species	Clone	Time CC1	Ventana Ultra validated dilution	Ventana Ultra validated incubationtime
CD003	Dako	A0452	Rabbit	Polyclonal	24 min CC1	1/150	32 min
CD008	Dako	M7103	Mouse	144B	32 min CC1	1/50	32 min
CD020cy	Dako	M0755	Mouse	L26	24 min CC1	1/500	16 min
Granzyme B	Monosan	MON7029-1	Mouse	GB7	32 min CC1	1/300 in Dako reduc dil	48 min
IBA-1	Wako Pure Chemical Industries, Ltd	019–19,741	Rabbit	Polyclonal	16 min CC1	1/4.000 in Dako reduc dil	32 min

*GFAP* Glial Fibrillary Acidic Protein, *EDTA* Ethylenediaminetetraacetic acid, *GAR* Goat-anti-rabbit, *GAM* Goat-anti-mouse, *biot* Biotinylated, *CD* Cluster of differentiation, *: part of the immunofluorescence triple staining, *cy* Cytoplasmic, *IBA-1* Ionized calcium-binding adapter molecule 1, *CC1* Cell conditioning 1, Dako reduc dil: antibody diluent, background reducing, catno. S3022, Dako

### Immunofluorescent triple labelling of CD3, CD8 and granzyme B

Sections were deparaffinised and antigen retrieval was performed as described above. After washing the sections thoroughly with phosphate-buffered saline (PBS), they were incubated with 5% normal goat serum at room temperature for 15 min. Then the CD3, CD8 and Granzyme B antibodies (Table 2) were diluted a 2% Bovine serum albumin/PBS block buffer simultaneously, and sections were then incubated in this combined antibody solution overnight at room temperature in a moist environment. Then, sections were washed thoroughly with PBS and incubated simultaneously with the concomitant fluorescently labelled secondary antibodies (Table 2) in a 2% Bovine serum albumin/PBS for 1 h in a moist dark environment. The sections were washed again with PBS and incubated with DAPI solution for 2 min, and briefly washed again in PBS. The sections were mounted using Prolong Gold Antifade Mountant (Thermo Fisher Scientific, Waltham, USA). Sections were scanned using the Vectra Polaris multispectral slide scanner (Perkin Elmer, Waltham, United States).

### Semi-quantitative analysis of CD3, CD8, CD20 and calbindin immunopositive cells.

To reduce investigator bias and ensure blinded manual counting, all slides were re-labelled with a new code by JB, who kept a key file. All sections were screened for immunopositive cells and photographed using brightfield illumination on a Leica DM5000B microscope with a Leica DFC 450 colour camera for brightfield (Leica, Wetzlar, Germany). Per patient, one section was used (CD3, CD8, CD20) and 10 regions of interest (ROI) were selected. Each ROI contained a longitudinal, and not curved, stretch of 900 µm consisting of white matter and all three anatomical layers of cerebellar grey matter (granular layer, Purkinje cell layer and molecular layer). For analysis of Calbindin, 20 ROI’s were selected, consisting each of a 900 µm longitudinal, and not curved, stretch of the Purkinje cell layer. These ROIs were analysed at a 10× magnification. Immunopositive cells, with a visible nucleus, were counted per morphological layer within these ROIs.

### Semi-quantitative analysis of microglial Iba-1 and TMEM119 immunopositive cells

Iba-1 immunoreactivity was semi-quantified by measuring the percentage of Iba-1 + staining in a section. Per patient one area of a section containing grey (granular, molecular and Purkinje cell layer) and white matter was selected (as seen in Fig. 3D–F) and analysed using ImageJ [17]. In ImageJ, the blue light frequency setting was selected to visualize the DAB stained Iba-1 + cells. A set black and white threshold was used to determine the percentage of Iba-1 immunopositive staining in the selected area.

Microglia can change their morphology upon a stimulus. They transform from a cell with a small soma and multiple ramifications into a cell with a large soma and few ramifications, Therefore, measuring soma area and total cell surface area and determining their ratio represents microglial status in an objective manner [18]. To this end, the outer surface area of Iba-1 + microglia, including ramifications, were encircled to estimate the total surface and the microglia’s soma was encircled to measure its surface. Iba-1 immunopositive cells were eligible for analysis if they were not overlapping with other microglial cells and at least the whole circumference of the soma and ramifications were visible. In one section per patient a total of 20 microglial cells were analysed using ImageJ, divided equally over the molecular layer, Purkinje cell layer, granular layer and white matter (five cells per layer) [17]. The ratio of the surface of the soma to the total surface of the microglia was calculated.

Since Iba-1 is also expressed by infiltrated monocytes/macrophages, we stained sections with TMEM119, a specific marker for microglia, to validate the immunoreactivity observed in the Iba-1 stained sections is indeed illustrating microglial identity. Iba-1 and TMEM119 immunoreactivity were compared in corresponding areas in adjacent stained sections.

### Statistical analysis

Statistical analyses were carried out using IBM SPSS Statistics for Windows version 26 (IBM Corp., Armonk, NY, USA).

As mentioned in the methods sections, the amount of CD3+ and CD8+ cells was counted per cerebellar layer, in 10 ROIs of a section per case. In some ROIs it occurred that no immunopositive cells were present in a specific layer, leading to a cell count of ‘0’. A cell count of ‘0’, also occurred in stretches of Calbindin immunopositive neurons in the Purkinje cells layer, mainly in NeuroCD patients. To account for the multiple counted values within a subject, groups were first overall compared with a generalised estimating equation. In the results section we address the estimated mean of counted cells per ROI per patient simple as cells per ROI per patient. In many areas ‘0’ positive cells were counted, therefore a negative binomial distribution was assumed with a logit link function to correct for the ‘0’ inflation. Subsequently, groups were pairwise compared by means of a Bonferroni post-hoc analysis by multiplying *p* values with 3, in order to account for the three different pairwise comparisons.

The percentage of Iba-1 immunoreactivity was expressed in median and interquartile ranges since the data was not normally distributed. Groups were compared using a Kruskal Wallis test.

Microglial status was determined by dividing the soma surface area by the total cell surface area. To compare groups we had to correct for multiple measurements within patients by means of linear mixed models, for which a random intercept for subjects was included. Groups were compared by means of a Bonferroni post-hoc analysis. *p* values < 0.05 were considered statistically significant. The confidence interval (CI) was 95%.

## Results

### NeuroCD patient description

Of the NeuroCD cases analysed, four were diagnosed with CD before and two after appearance of neurological symptoms according to standard diagnostic criteria [19]. Of these cases, three were diagnosed with refractory CD type 2 (RCD) and one patient developed an enteropathy associated T-cell lymphoma. A detailed description of the medical history per patient is provided in the Additional file 1.

### NeuroCD is associated with atrophy and loss of Purkinje cells

Upon both macroscopic and microscopic examination of the sections, cortical atrophy was apparent in both the NeuroCD and SCA cerebella. This was characterised by slender folia and larger spaces between cerebellar folia in SCA and NeuroCD cases when compared to NNC cases (Additional file 1: Fig. S1) [14]. This could be attributed to a markedly decreased cell density in the molecular layer and granular layer of the cerebellum in SCA and NeuroCD patients.

**Fig. 1:**
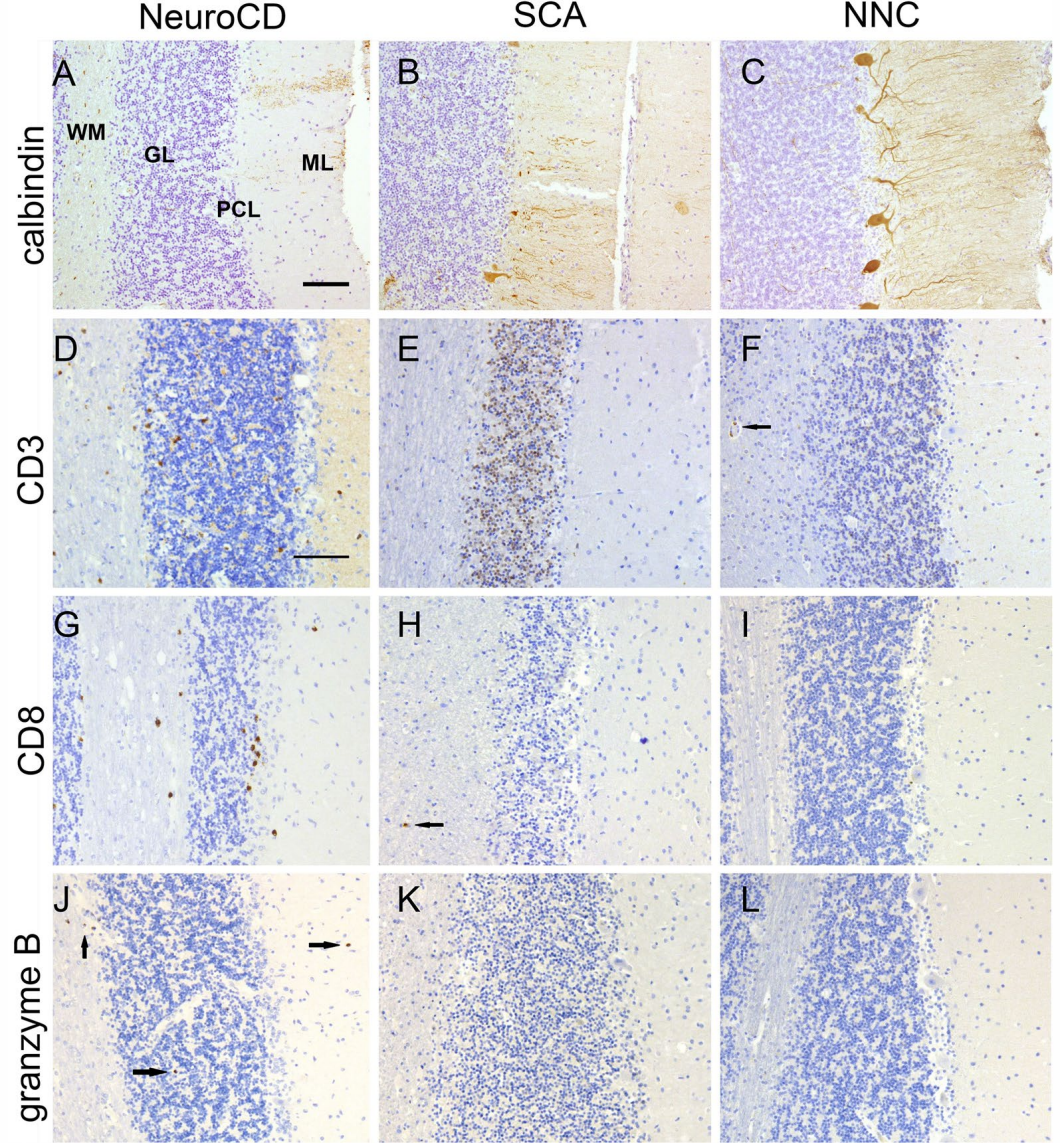
Brightfield microscopy pictures taken per group of histopathological staining performed on formalin-fixed paraffin embedded cerebellar tissue sections. **A**, **D**, **G**, **J** represent cerebellar tissue of a neurocoeliac disease patient (NeuroCD), **B**, **E**, **H**, **K** is representative of a spinocerebellar ataxia patient (SCA) and **C**, **F**, **I**, **L** of a non-neurological control (NNC). **A**–**C** demonstrate the Calbindin staining for Purkinje cells, which are still apparent in the non-neurological controls, but have largely disappeared in NeuroCD and SCA. **D**–**F** shows CD3 staining. Multiple CD3 + cells are visible in all layers of the cerebellar tissue in NeuroCD, especially in the granular layer and the Purkinje cell layer. In SCA CD3 + cells are absent. In NNC just two CD3 + cells are visible in a blood vessel in the white matter indicated by an arrow. **G**–**I** demonstrate CD8 + cells, which are abundant in NeuroCD, especially in de Purkinje cell layer, whereas in SCA only one CD8 + cell is spotted (indicated by an arrow) and no CD8 + cells are found in NNC. **J**–**L** exhibits the Granzyme B staining, which is (scarcely) apparent in NeuroCD in all layers (indicated by black arrows), but is absent in SCA and NNC Tissue was counterstained with haematoxylin, Scale bar (**A**–**L**): 100 um. *WM* White matter, *GL* Granular layer, *PCL* Purkinje cell layer, *ML* Molecular layer

The suspected loss of Purkinje cells was substantiated by a dramatic reduction in Calbindin immunoreactivity in NeuroCD cases. This decrease was even more prominent than in cases with SCA, a disease marked by pervasive Purkinje cell degeneration (Fig. 1A–C and Table 4). By semi-quantitative analysis, we found a mean decrease in Purkinje cells in NeuroCD of 81.3% in comparison to NNC, whereas the Purkinje cell loss in SCA patients was 58.6% compared to NNC. The loss of Purkinje cells was accompanied by Bergmann gliosis as illustrated in Additional file 1: Fig. S2.

**Table 4:** The estimated mean cell count per area per patient comparing cell count in different cerebellar layers between groups

IHC staining	Layer	NeuroCD	CI lower	CI upper	Spino-cerebellar Ataxia	CI lower	CI upper	Non-neurological control	CI lower	CI upper	Bonferroni P NeuroCD_vs_SCA	Bonferroni P NeuroCD_vs_NNC
CD3	Molecular Layer	2.75	1.61	4.69	0.14	0.07	0.27	0.57	0.27	1.18	**0.001**	**0.015**
	Purkinje Cell Layer	4.57	2.77	7.54	0.24	0.07	0.81	0.87	0.36	2.09	**0.001**	**0.008**
	Granular Layer	3.23	1.97	5.31	0.36	0.16	0.83	0.77	0.25	2.32	**0.002**	**0.023**
	White Matter	3.42	1.96	5.97	0.54	0.21	1.42	0.52	0.22	1.21	**0.013**	**0.011**
	Perivascular	2.00	1.19	3.35	0.72	0.41	1.27	0.43	0.29	0.65	**0.072**	**0.010**
	TOTAL COUNT	**15.97**	9.76	26.13	**2.00**	0.96	4.17	**3.15**	1.53	6.48	**0.002**	**0.006**
CD8	Molecular Layer	1.35	0.70	2.61	0.20	0.14	0.30	0.08	0.04	0.16	**0.035**	**0.016**
	Purkinje Cell Layer	2.30	1.18	4.47	0.10	0.06	0.17	0.08	0.03	0.23	**0.015**	**0.014**
	Granular Layer	2.23	1.07	4.65	0.06	0.03	0.12	0.18	0.09	0.38	**0.028**	**0.043**
	White Matter	4.02	1.99	8.11	0.36	0.16	0.81	0.08	0.04	0.20	**0.035**	**0.019**
	TOTAL COUNT *	**12.35**	6.29	24.26	**1.16**	0.79	1.70	**0.92**	0.39	2.15	**0.026**	**0.022**
Calbindin		1.13	0.61	2.11	2.50	1.51	4.15	6.04	5.14	7.10	**0.194**	**0.000**

The total count is a summation of all layers. A generalised estimating equation was performed with a post-hoc Bonferroni to correct for the different measurements within patient groups. * Including perivascular cells, *CI* Confidence interval was 95%, upper and lower limit are indicated per measurement. *p* values < 0.05 were considered statistically significant. *IHC* Immunohistochemistry, *NeuroCD* Neurocoeliac disease, *SCA* Spinocerebellar ataxia, *NNC* Non-neurological controls

### *Infiltration of CD3*+ *, CD8* + *and granzyme B* + *cells in neuro CD patients*

**Fig. 2:**
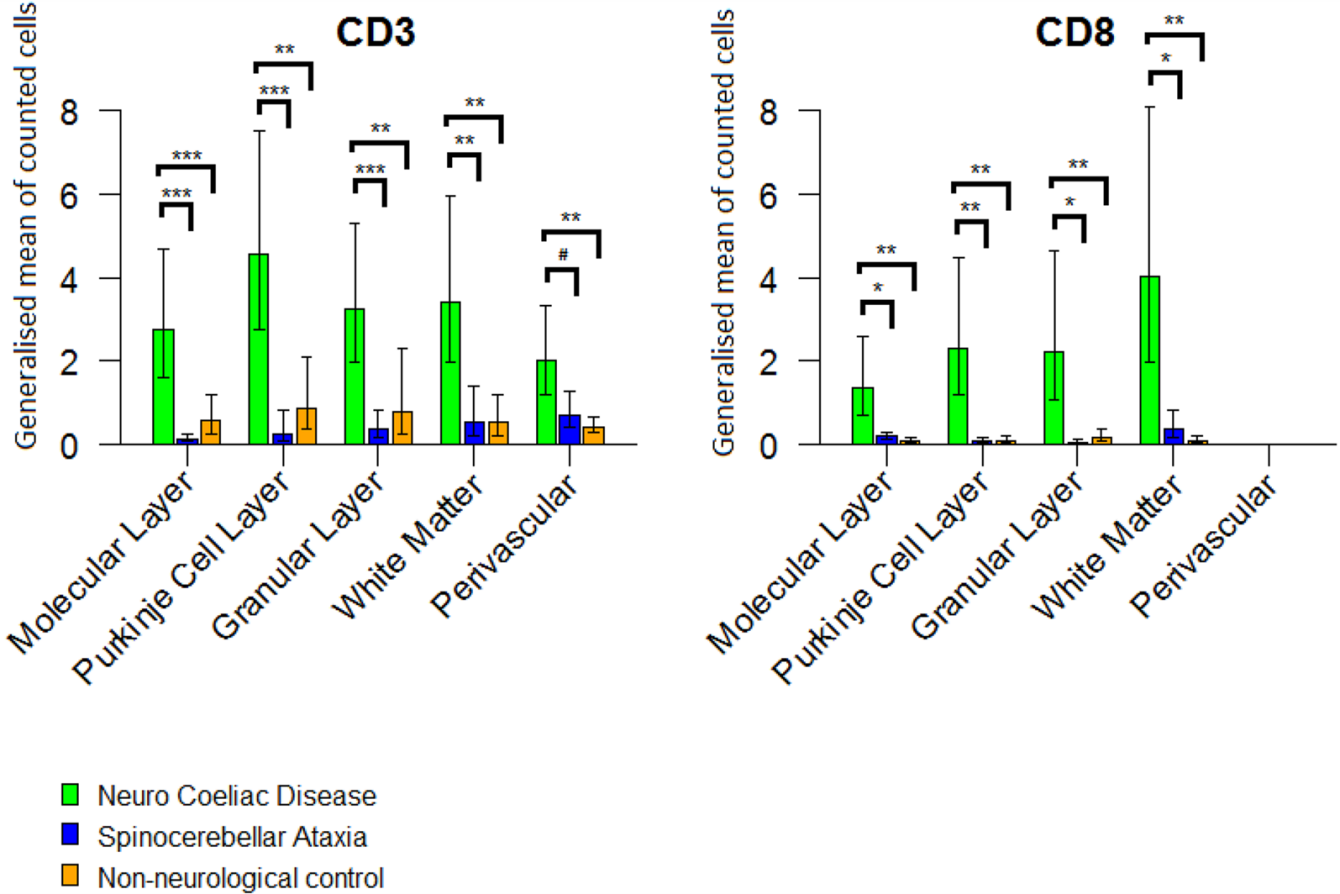
The bars in the CD3 and CD8 graph display the generalised estimate of the mean of cells counted per area per layer of the cerebellum per patient (y-axis). The lines display the lower and upper limit of the confidence interval (95%). The green colour represents Neuro Coeliac Disease, the blue colour represents Spinocerebellar ataxia and the orange colour represents Non-neurological controls. Significance is indicated in the following manner: * = p < 0.05, ** = p < 0.01, *** = *p* < 0.001

Whereas presence of CD3+ T-cells was observed in cerebella of all groups (Fig. 1D–F), by far most CD3+ immunoreactivity was found in NeuroCD cases (15.97 cells per ROI per patient), versus only 2.00 (*p* = 0.002) CD3+ immunopositive cells in SCA and 3.15 (*p* = 0.006) CD3+ cells in NNC cases (Fig. 1D–F, Fig. 2A and Table 4). In NeuroCD, more CD3+ immunoreactivity was observed in the grey matter (10.55 cells per region) compared to the white matter (3.42 cells) (Fig. 2A). In SCA a similar amount of immunopositive cells of 0.74 and 0.54 was found in grey and white matter, respectively. Overall, the CD3+ immunoreactivity in SCA cases was very low, similar to NNC (2.21 and 0.52 immunopositive cells in grey and white matter, respectively) (Fig. 1 E, Fig. 2A). Interestingly, in NeuroCD, most CD3+ immunoreactivity was observed in and around the Purkinje cell layer (4.57 cells per region). This is over five times more than in NCC and over 19 times more than in SCA (respectively CD3+ cell count per area, per patient: 0.87, 0.24) (Fig. 2A, Table 4). Some CD3+ cells were also observed intra- or perivascular in both grey and white matter in all groups.

Since we observed a remarkable CD3 reactivity near the Purkinje cell layer and a significant loss of Purkinje cells in the NeuroCD group, we hypothesized that cytotoxic CD8 T-cells are responsible for the Purkinje cell destruction [20].

We observed sparse CD8 immunoreactivity in both SCA and NNC cases (Fig. 1G, H). In NeuroCD the CD8+ cell count was about twelve times higher compared to controls (Fig. 2B, Table 4). More CD8 immunoreactive cells were counted in the grey matter compared to white matter. Within the grey matter, CD8+ cells were concentrated in the Purkinje cell layer and the granular layer, and relatively dispersed in the molecular layer. These observations imply an infiltration of potentially cytotoxic CD8 T-cells to areas with marked neuronal cell loss. When comparing the refractory CD cases with the regular CD patients, similar CD3+ and CD8+ cell numbers were observed (19.47 CD3+ -cells and 15.23 CD8+ cells in RCD and 12.37 CD3+ cells and 9.47 CD8+ cells in regular CD, not significant).

The clear increase in CD8+ cells suggests cytotoxic T-cell activity. To establish this we analysed sections for Granzyme B immunoreactivity. In NeuroCD patients Granzyme B positive cells were scarcely exhibited in different cerebella, indicating an ongoing cytotoxic process (Fig. 1J). This Granzyme B immunoreactivity was neither observed in the SCA patients nor in NNC (Fig. 1K, L).

**Fig. 3:**
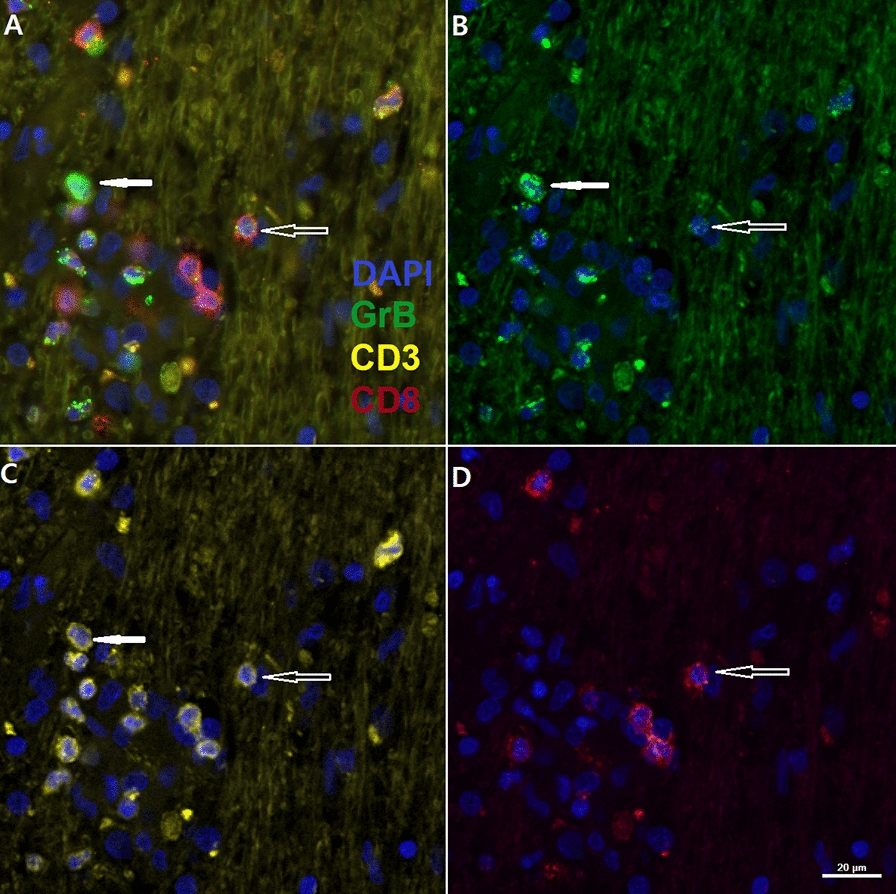
Depicts co-localization of CD3, CD8 and Granzyme B in the cerebellar white matter of a NeuroCD patient. **A** is an overlay of CD3 in yellow, CD8 in red, Granzyme B (GrB) in green, and DAPI in blue. **B** depicts Granzyme B immunoreactivity, **C** depicts CD3 immunoreactivity and **D** depicts CD8 immunoreactivity, all with DAPI in blue. Multiple CD3 + /CD8 + cells are visible as well as triple positive cells. The white arrow indicates a CD3 + /CD8-/Granzyme B + cell. Scale bar: 20um

A triple staining technique was performed to determine co-localisation of CD3, CD8 and Granzyme B in NeuroCD. The selected NeuroCD sections displayed triple positive cells in both white matter and grey matter. Figure 3 depicts infiltration of CD3+/CD8+/Granzyme B+ cells in white matter indicated by the hollow white arrow. Sporadically CD3+/CD8-/Granzyme B+ cells were seen (Fig. 3 indicated by the solid white arrow), possibly reflecting natural killer-T-cells.

### *Absence of CD20*+ *B-cells in NeuroCD*

CD20 B-cells were extremely rare, therefore we did not select regions of interest, but instead the entire cerebellar section was analysed. In each group there were two patients without any CD20+ cells, the median cell count in NeuroCD was 3 cells, SCA 2 cells and NNC 6 cells. The CD20+ cell count did not vary between groups.

### Changes in astroglial and microglial appearance in NeuroCD

**Fig. 4:**
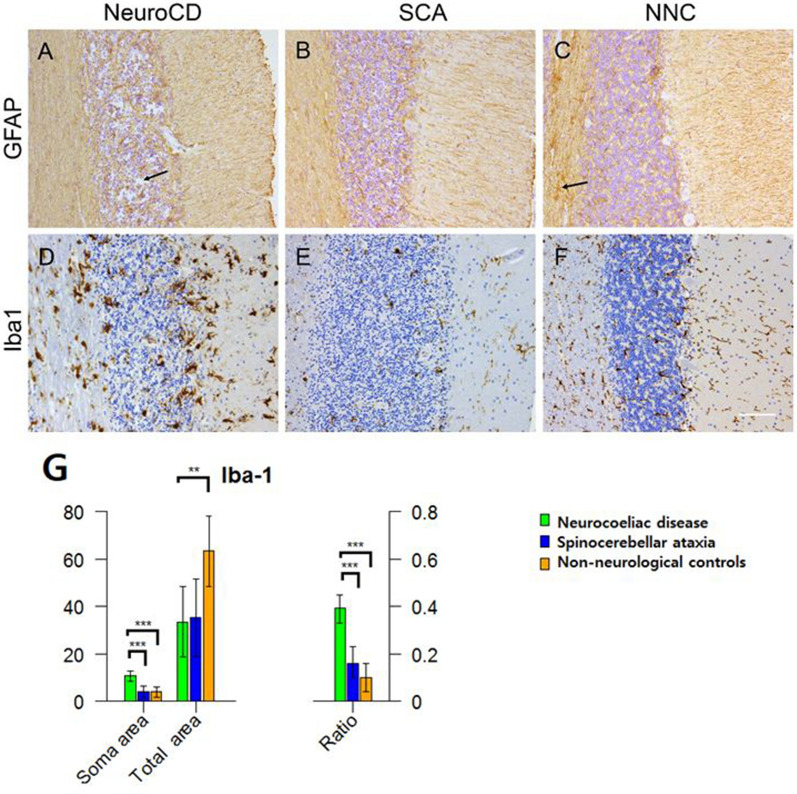
Brightfield microscopy pictures taken per group of histopathological staining performed on formalin-fixed paraffin embedded cerebellar tissue sections. **A**–**C** exhibit the GFAP staining. **A** exhibits a disorderly distribution of GFAP filaments with occasional thickening of branches in NeuroCD and a loss of neurons in the granular layer. B and C exhibit a higher cell density in SCA and NNC compared to NeuroCD. The black arrow in A indicates the granular layer with a clear loss of density of GFAP immunoreactivity and disintegrated tissue due to loss of neurons. The black arrow in C points to increased GFAP immunoreactivity. **D**–**F** exhibit Iba-1 staining. Numerous iba-1 positive cells can be found in NeuroCD and NNC patients, while less in SCA. **D** Iba-1 positive cells in NeuroCD have a large soma compared to those in SCA and NNC. Scale bar (**A**–**F** 100 um. **G** The graph on the left side displays the generalized estimate of the mean Iba-1 positive soma surface area and the total cell surface area (*Y*-axis displays surface in µm^2^). The graph on the right represents the ratio) of the latter (displays the generalized estimate of the mean soma surface area: the total cell surface area). In both graphs the lines display the lower and upper limit of the confidence interval (95%). In the Neurocoeliac disease group a relatively large soma surface was measured and a relatively small total cell surface, leading to a higher ratio

Glial Fibrillary Acidic Protein (GFAP) was used as a marker for astrocytes. In NeuroCD patients a disorderly distribution of GFAP immunoreactive filaments was observed compared to a more regular distribution in SCA and NNC cases (Fig. 4A–C). Compared to the NNC cases less GFAP immuno-reactivity was observed in NeuroCD patients, in the molecular layer, and even more apparent in white matter. A clear loss of density of GFAP immunoreactivity in the granular layer is depicted in Fig. 4A, indicated by a black arrow, while Fig. 4C shows increased GFAP expression indicated by the black arrow. In SCA subjects there was less GFAP immunoreactivity observed as well, especially in white matter (Fig. 4B).

Due to the background staining these findings were not quantifiable.

Microglial cells are the resident immune cells of the brain and are responsive to damage or pathogens, by changing their morphology and function. In a homeostatic state they scan the area surrounding them with their strongly ramified branches [21]. Upon encountering a pathogen or injury, they alter their morphology, and may release inflammatory cytokines, neurotrophic factors and growth hormones, and are able to clear apoptotic cells [22]. We quantified the presence of microglial cells by their expression of Iba-1 immunoreactivity. Iba-1 immunoreactive cells were most abundant in Neuro-CD (Fig. 4D). The percentage of Iba-1 immunoreactivity observed in NeuroCD (median: 8.5%, interquartile range (IQR): 5.55) was the highest, followed by NNC (median: 3.4%, IQR: 4.93) and SCA (median: 3.4%, IQR: 6.31). However, this difference was not significant between NeuroCD and NNC (*p* = 0.055), nor for NeuroCD and SCA (*p* = 0.100).

In addition to cell count we also analysed the distribution and morphology of these cells as this reflects the activation state of these cells. In NNC cases, Iba-1 + microglial cells were evenly distributed in the cerebellar layers and mostly had a ramified morphology with a small soma and slender spread out branches (Fig. 4F). The mean analysed soma surface was 3.9 µm^2^, with a total cell surface of 63.4 µm^2^ (Fig. 4G). The Iba-1 + cells in NeuroCD (Fig. 4D) had a different morphology in comparison to the Iba-1 + cells in NNC and SCA (Fig. 4E, F). Most microglial cells observed in NeuroCD were characterised by a swollen, larger soma and shorter, truncated processes. The mean soma surface in was NeuroCD 10.6µm^2^, with a relatively small total cell surface of 33.5 µm^2^ (Fig. 4G). In SCA a variety was seen in morphology, ranging from an amoeboid to a ramified shape of microglia (Fig. 4E). The soma surface in SCA subjects was 4.1 µm^2^, and the total cell surface was 35.2 µm^2^ (Fig. 4G). So, in NeuroCD patients the soma area was larger compared to SCA patients and NNC (*p* < 0.001) and the total cell area was smaller compared to the NNC subjects (*p* < 0.01) (Fig. 4G). The ratio of soma surface area and total microglial cell surface area was highest in NeuroCD patients (*p* < 0.001), indicating a reactive state.

**Fig. 5:**
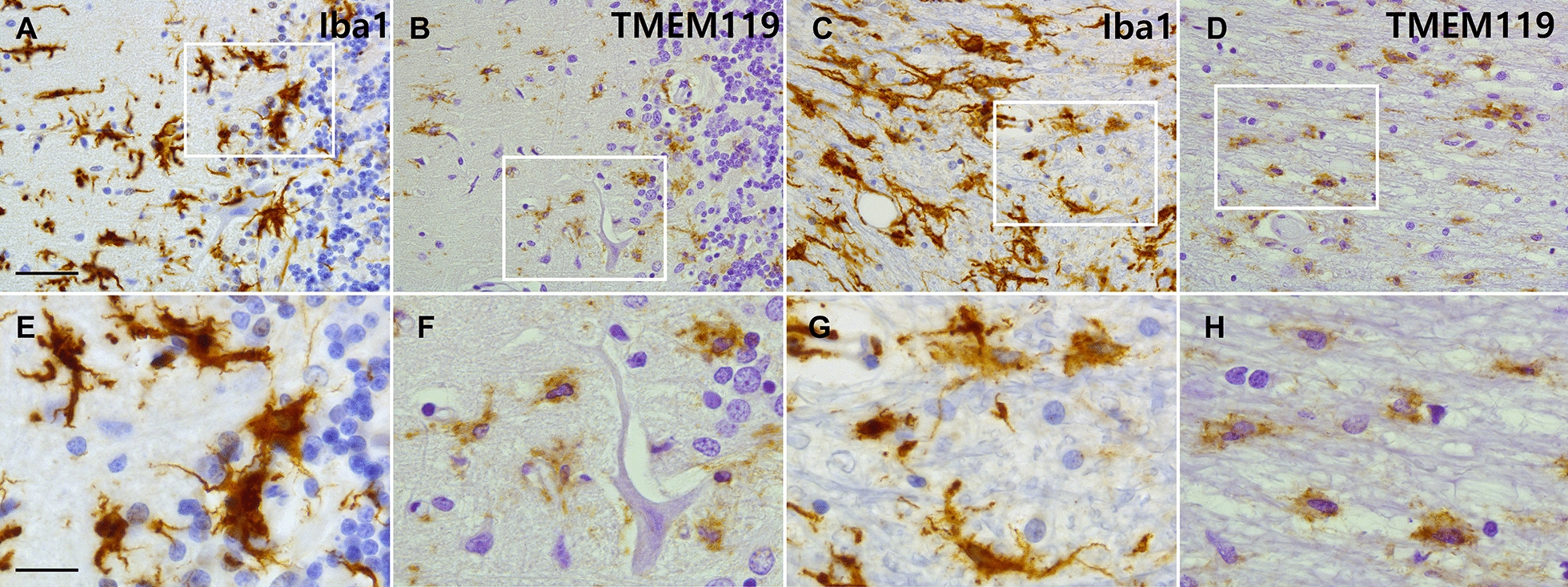
Shows Iba-1 and TMEM119 immunoreactivity in sequentially cut sections of a neurocoeliac disease patient. **A** and **B** focus on the grey matter and specifically on the Purkinje cell layer, showing Iba-1 and TMEM119, respectively. **C** and **D** focus on the white matter, Iba-1 staining in **C** and TMEM119 in **D**. Lower panels represent higher magnifications of the white rectangles in the top panels. Figure **F** shows a remaining Purkinje cell. A similar immunoreactivity pattern can be seen in both grey matter and white matter while comparing Iba-1 and TMEM119, establishing that the Iba-1 immunoreactivity truly portrays microglia. Scale bar figure **A**–**D** 50 um, Figure **E**–**H** 20 um

In order to validate that Iba-1 immunoreactivity observed indeed represents microglia and not infiltrating monocytes/macrophages, we then stained sections with a specific marker for microglia; TMEM119. We selected corresponding areas within individual sections to compare Iba-1 and TMEM119 immunoreactivity. As portrayed in Fig. 5Iba-1 immunoreactivity is matched by TMEM119 reactivity in grey matter (Fig. 5A, B, E, F) as well as white matter (Fig. 5C, D, G, H), validating their microglial identity.

## Discussion

In the present study we analyzed both Purkinje cell degeneration and the presence of innate and adaptive immune cells in the cerebellum of a cohort of NeuroCD cases compared to SCA and NNC subjects. Cerebellar atrophy with a thinning out of the molecular layer and granular layer (due to a loss of neurons with disorderly reactive gliosis) was observed in NeuroCD. A dramatic loss of Purkinje cells coinciding with increased CD3 + lymphocyte infiltration, the presence of Granzyme B + cytotoxic lymphocytes and an altered morphological phenotype of local microglial cells was observed. Alongside this loss of Purkinje cells some Bergmann gliosis was detected. In contrast to previous case studies on histopathological findings in NeuroCD patients [14–16, 18], we could semi-quantitatively analyse cerebellar post-mortem material from multiple cases which strengthens our study. We are the first to observe microglial reactivity in cerebellum of NeuroCD patients. Moreover, a comparison to SCA patients was performed in which a genetic predisposed loss of Purkinje cells occurs [23]. In several forms of SCA the demise of Purkinje cells is caused by the formation of aggregates of the mutated ataxin-1 protein [24]. In paraneoplastic cerebellar degeneration more and more evidence of a T-cell mediated Purkinje cell death is accumulating [25]. The high ratio of CD8 + lymphocytes observed in or near the Purkinje cell layer of our NeuroCD cohort also implies a T-cell mediated mechanism, correlating with the intestinal damage in CD. Purkinje cells are able to upregulate MHC class I expression during an inflammatory process, which may provide the opportunity for CD8 + lymphocytes to recognize antigens presented by Purkinje cells and lyse these neurons by excretion of Granzyme B and perforin [26].

Indeed, CD8+ and Granzyme B+ cells were mainly found in NeuroCD. Ligation of the T-lymphocyte receptor initiates the release of Granzyme B, enabling the lymphocyte to induce apoptosis in many target cells [27].

Interestingly, relatively more lymphocytes were found in the Purkinje cell layer in NeuroCD patients compared to the other groups, even in areas where Purkinje cells have disappeared and gliosis has taken over. There was a small interpatient variation in the NeuroCD group in lymphocyte count and, to a lesser extent, in microglial reactive status. Two patients (NeuroCD 5 and 6) had the lowest cell counts and the least microglial reactivity. This might be explained by the extent of their neurological disease course. These patients suffered from neurological complaints until death for a long period; respectively 24 and 13 years (Table 1). In NeuroCD patients that experienced a short period of neurological disease before they died, relatively more immune cells were observed. It could be hypothesized that over this longer period of neuro-inflammation, the neuro inflammatory process is extinguished after the extermination (or disappearance) of the target antigen. In our statistical analysis we have taken this small interpatient variation into account.

In NeuroCD patients we observed a reactive state of microglia contrary to a more homeostatic state in NNC with a small soma and a more ramified appearance. Microglia are the main cerebellar innate immune cells involved in surveillance of the brain. Upon damage, microglia change their phenotype and can act as antigen presenting cells or phagocytes and release inflammatory cytokines, neurotrophic factors and growth hormones exerting both protective and detrimental effects [28, 29]. In several SCA animal models microglia were found to actively contribute to the neuropathological process depending on the stage of disease. In SCA-1 for example increased microglial reactivity and TNF-α production is observed preceding neuro-inflammation and loss of Purkinje cells [30]. We therefore propose that microglia reactivity in NeuroCD may reflect their role in neuropathology instead of exerting neuroprotective effects.

Our results suggest a role for T-lymphocytes as well as microglia in the pathological process contributing to the neurological complications, although the exact target antigen for this immunological cell recruitment is yet to be found. Earlier studies have suggested transglutaminase 2 and 6 as potential antigens. Transglutaminase 2 is the main auto antigen in CD and has been hypothesized to alter the blood brain barrier [16]. The possible role of transglutaminase 6 in NeuroCD is still controversial. It has been detected in cerebellar tissue of gluten ataxia patients, and its antibodies have also been detected in other neurological diseases (amyotrophic lateral sclerosis [31], multiple sclerosis [32], spinocerebellar ataxia type 35 [33], cerebral palsy [34], schizophrenia [35]). However, the detection of transglutaminase 6 in cerebellar tissue in these studies was neither limited to gluten sensitive patients (serological detection of anti-gliadin antibodies) nor to CD patients.

Based on our findings, another possible mechanism driving the neurological damage might be the transformation of lymphocytes as seen in RCD2. At least half of the NeuroCD patients in our cohort had RCD2 and at least one had developed an enteropathy-associated T-cell lymphoma. Refractory coeliac disease (that can be seen as a pre-malignant condition) and EATL might be risk factors for developing cerebellar complications in themselves, as this association has been described before [36–40]. Similar to previous cases, the NeuroCD patients were 50–75 years-old at onset of neurological symptoms, indicating that NeuroCD might be a late onset presentation of CD or a late complication. Both RCD and NeuroCD are more commonly diagnosed in elderly patients, and CD diagnosis later in life is associated with a more severe disease course [41–43]. Whether this enhanced response is related to prolonged gluten exposure before CD diagnosis is unknown. [44, 45]. While neuropathies and other neurological disorders have often been attributed to malabsorption in untreated CD, this was not the case in our cohort. Our neuropathological findings clearly point in the direction of an (auto-)immune mediated mechanism. It is tempting to speculate that the refractory character of CD in these cases might also explain why NeuroCD patients develop progressive neurological complaints despite adhering to a gluten-free diet.

This study has several limitations that should be mentioned. As cerebellar tissue was provided by different institutions, different protocols were followed for preparation and fixation of cerebellar tissue. However this did not form a barrier for immunohistochemistry, which was performed successfully on all tissue sections. We are aware that our observations were done in post-mortem cerebellum of a limited number of NeuroCD patients of which we were able to obtain brain material from. This material is rare and therefore we were not in the situation to select cases, which prevents selection bias.

This study was limited to the cerebellum, however a broader diffuse inflammatory process and involvement of other parts of the nervous system is very likely. Unfortunately we had no access to duodenal tissue and limited access to serum, and DNA extraction from the fixed cerebellar tissue was not sufficient. This made it impossible to determine the HLA-DQ status or to examine T-cell receptor clonality.

In conclusion, we have demonstrated significantly higher CD3+ and CD8+ lymphocyte counts, exhibiting Granzyme B activity and a demise of Purkinje cells in the cerebellar tissue and of NeuroCD compared to controls. We were the first to investigate the phenotype of microglia in NeuroCD. Microglial cells show an activated morphology in all cerebellar layers in NeuroCD. These findings together strongly suggest an immune cell mediated form of neurodegeneration in NeuroCD. The next step would be to unravel the signalling factors underlying the neuropathological mechanism, paving the way for targeted drug therapies in this rare but severe disease.

## Supplementary Information

**Additional file 1.** A detailed patient description.

## Data Availability

The datasets generated during the current study are not publicly available, but are available from the corresponding author on reasonable request.

## Abbreviations

AGA: Anti-gliadin antibodies
CD: Coeliac disease
GFAP: Glial fibrillary acidic protein
IEL: Intraepithelial lymphocytes
NBB: Netherlands Brain Bank
NeuroCD: Neuro-coeliac disease
NNC: Non-neurological controls
PBS: Phosphate-buffered saline
PC: Purkinje cells
RCD: Refractory coeliac disease
ROI: Region of interest
SCA: Spinocerebellar ataxia
TBS: Tris-buffered saline
