# Supplementary material for: Cerebellar presence of immune cells in patients with neuro-coeliac disease
Source: Acta Neuropathol Commun. 2023 Mar 25;11:51. doi: 10.1186/s40478-023-01538-5 (PMC10040112; doi:10.1186/s40478-023-01538-5)
Supplement: Supplementary file 1 — Additional file 1. A detailed patient description. [file 40478_2023_1538_MOESM1_ESM.docx]

**Supplementary data**

**Patient description**

NeuroCD1: Male patient, diagnosed with CD at the age of 49 years. He had a family history of CD. Vitamins, phosphate and ferritin values were within normal range. Despite the initiation of a strict GFD, the patient still experiences fatigue and arthralgia five years later. A repeat biopsy demonstrated subtotal villous atrophy and a further increase of intra-epithelial lymphocytes. He received thiopurines (200mgs daily) and cladribin, despite of this treatment an increase in intra-epithelial lymphocytes was observed as well as villous atrophy, leading to the diagnosis RCD2. Neurological complaints (starting with diplopia and a disturbed coordination) came to light nine years later. Based on monoclonal T-cell receptor gene rearrangements in a duodenal biopsy an enteropathy-associated T-cell lymphoma (EATL) was diagnosed and the patient was admitted for autologous stem cell therapy. His neurological status further deteriorated and a brain biopsy was performed. An infiltrate of CD3+/CD8+/Granzyme B+ cells was found. A T-cell receptor gene rearrangement assay demonstrated a possible peak, however not compatible to the peak found in duodenal tissue. Clinical information did not mention any (overt) alcohol use. Additional neuropathological findings: No signs of Parkinson’s disease, Alzheimer’s disease or vascular disease.

NeuroCD2: Female patient, she suffered of diarrhoea since childhood. In the months before her neurological complaints started, the diarrhoea intensified and the patient lost a lot of weight. Enteral nutrition through a duodenal tube was started, without effect on the diarrhoea and the weight loss. Therefore parenteral nutrition was initiated. A duodenal biopsy demonstrated villous atrophy (Marsh 3C) and monoclonal T-cell receptor gene rearrangements, she is a carrier of HLA-DQ8, leading to the diagnosis RCD2. Based on histopathological findings and the presence of aberrant cells (cytoplasmicCD3+, CD30+, CD4- CD8-). She developed a cerebellar syndrome and myoclonus. A muscle biopsy (m. tibialis antirior fine needle biopsy) yielded some CD8+ cells. Clinical information did not mention any (overt) alcohol use. Additional neuropathological findings: No signs of Parkinson’s disease, Alzheimer’s disease or vascular disease.

NeuroCD3: Female patient, already known with CD, polymyalgia rheumatica and thyroid disease when neurological complaints started at the age of 69 years. She suffered from myoclonus and cerebellar syndrome. Both progressed rapidly and were soon accompanied by apathy and moodswings. The cerebellar ataxia progressed rapidly in two years’ time. She was treated with methylprednisolone without result and dead at the age of 71. Clinical information did not mention any (overt) alcohol use. Additional neuropathological findings: staining was negative for amyloid, Tau, alpha-synucleine. No signs of Parkinson’s disease, Alzheimer’s disease or vascular disease.

NeuroCD4: Male patient, diagnosed with CD previous to the start of neurological complaints and adhered to a gluten-free diet. At the age of 75 years he developed a cerebellar syndrome with progressive (extra-) pyramidal symptoms. He lost a lot of weight and passed away two years later. Clinical information did not mention any (overt) alcohol use. Additional neuropathological findings: staining was negative for alpha-synucleine. A few diffuse amyloid plaques were seen, reviewed as normal for the patients age by the neuropathologist. No signs of Parkinson’s disease, Alzheimer’s disease or vascular disease.

NeuroCD5: Female patient that was initially examined due to excessive weight loss; a duodenal biopsy portrayed total villous atrophy (Marsh3C). Subsequently she was diagnosed with CD, which proved to be non-responsive to a gluten-free diet. Repeated testing for TGA2 was <1, repeat duodenal biopsy demonstrated a total villous atrophy, crypt hyperplasia, loss of CD8+ cells and clonal T-cell beta, gamma and delta-chain gene rearrangements on multiplex PCR and she was diagnosed with RCD2. An EATL was ruled out. She was treated with azathiopurine 50mg and prednisone 40mg which positively affected her weight. However, her ataxia (which was present from the start) kept on progressing. Vitamin b12, folate and ferritin values were all normal. She underwent autologous stem cell transplantation with minor effect. She had a family history of CD and multiple sclerosis. Clinical information did not mention any (overt) alcohol use. No signs of Parkinson’s disease, Alzheimer’s disease or vascular disease.

NeuroCD6: female patient, diagnosed with CD at the age of 50 years. Concomitant vitamin B12, folic acid and iron deficiencies were treated effectively and a gluten-free diet was initiated. In the following two years a slowly progressive cerebellar syndrome became apparent with abnormal body movements and myoclonic jerks. In 1994 she was diagnosed with CD triggered Ramsay-Hunt cerebellar syndrome in absence of other possible causes. She underwent treatment with prednisone without effect. She developed severe dysarthria, disorderly behavioural, depression, she abandoned her gluten-free diet and lost a lot of weight (19 kg). eventually she developed respiratory failure and passed away. The detailed clinical history did not mention alcohol use or abuse. Additional neuropathological findings: No Lewy bodies were seen. No signs of Parkinson’s disease, Alzheimer’s disease or vascular disease .Supplementary figure 1


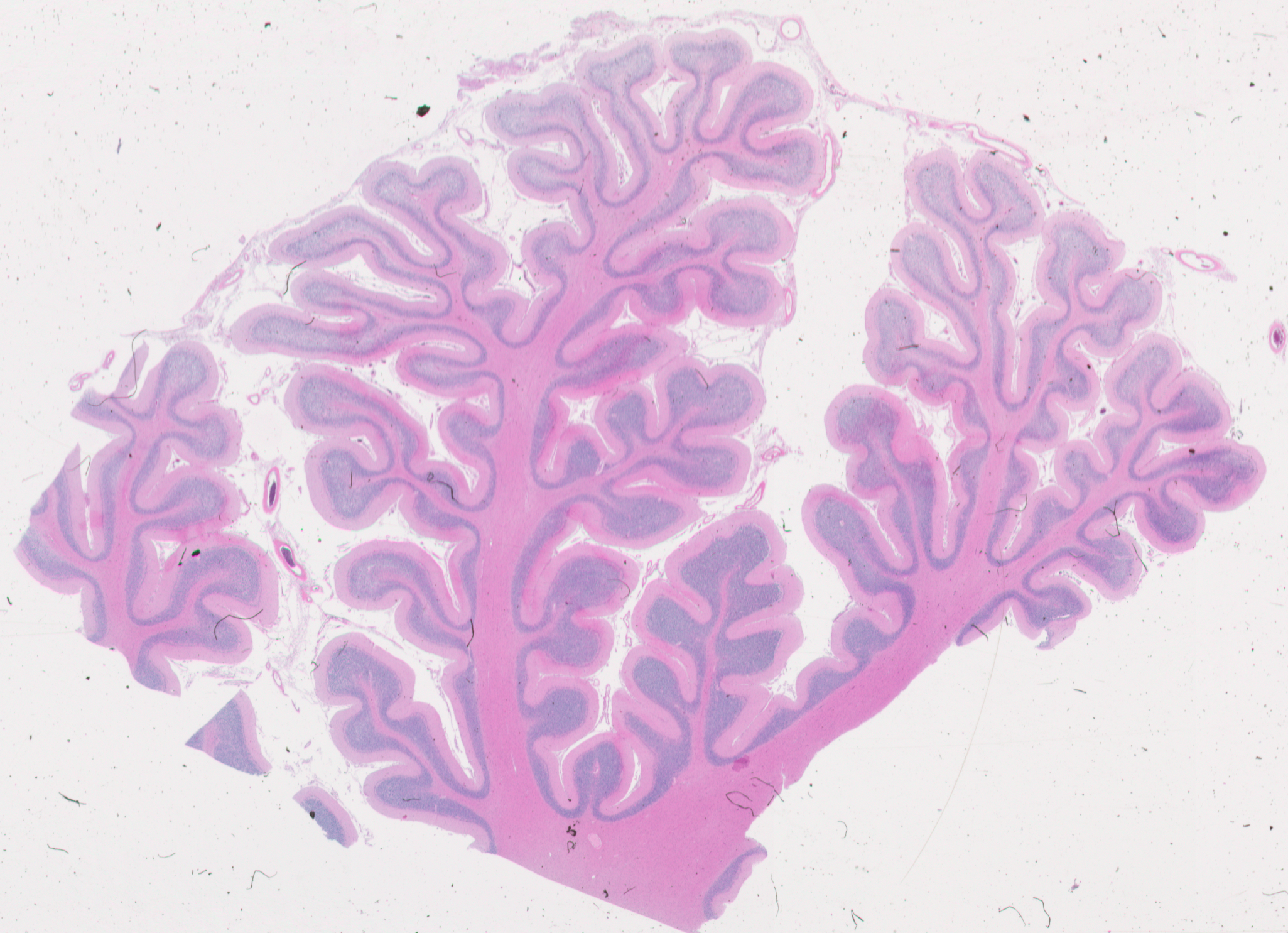


Figure 1 shows a fullsize overview of the vermis of a NeuroCD patient. Severe atrophy can be observed with a thinning out of both the granular layer and molecular layer. H&E stain.

Supplementary figure 2


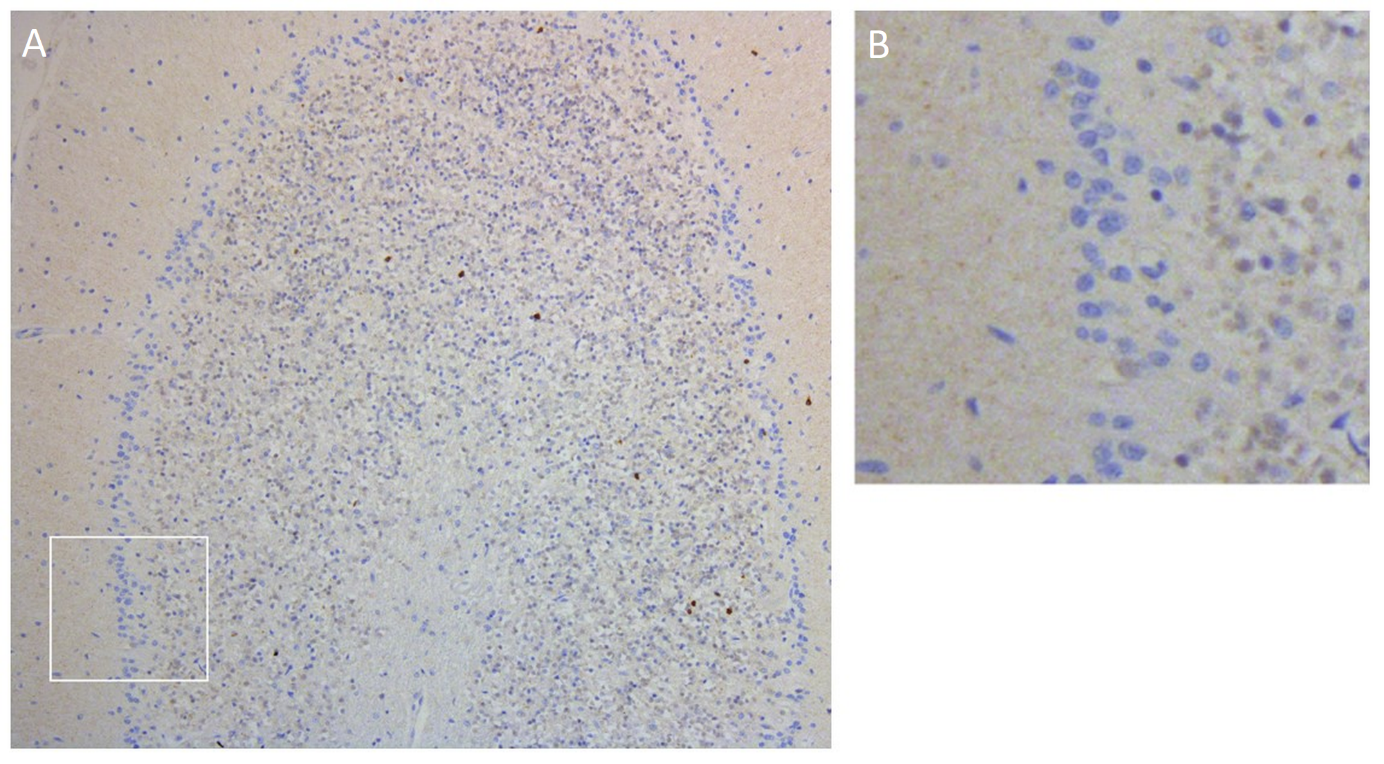


Figure 2 A shows a close up of a folium of the vermis of a NeuroCD patient with loss of Purkinje cells and hyperplasia of Bergmann glia throughout the Purkinje cell layer. Figure 2 B is a magnification of the white frame in figure A, highlighting the Bergmann gliosis, the larger blue stained cells running through the middle in the Purkinje cell layer and absence of Purkinje cells. H&E + CD3 counterstain.
